# Premature adrenarche and metabolic risk: a systematic review and meta-analysis

**DOI:** 10.1093/ejendo/lvaf167

**Published:** 2025-08-30

**Authors:** Wogud Ben Said, Ioannis G Lempesis, Silvia Fernandez-Garcia, Shakila Thangaratinam, Wiebke Arlt, Jan Idkowiak

**Affiliations:** Centre for Endocrinology, Diabetes and Metabolism, Birmingham Health Partners, University of Birmingham, Birmingham, B15 2TT, United Kingdom; Department of Endocrinology and Diabetes, Birmingham Children's Hospital, Birmingham Women's and Children's NHS Foundation Trust, Birmingham, B4 6NH, United Kingdom; Department of Metabolism and Systems Science, School of Medical Sciences, College of Medicine and Health, University of Birmingham, Birmingham, B15 2TT, United Kingdom; NIHR Birmingham Biomedical Research Centre, Women's Metabolic Health Theme, University of Birmingham, Birmingham, B15 2TT, United Kingdom; Centre for Endocrinology, Diabetes and Metabolism, Birmingham Health Partners, University of Birmingham, Birmingham, B15 2TT, United Kingdom; Department of Metabolism and Systems Science, School of Medical Sciences, College of Medicine and Health, University of Birmingham, Birmingham, B15 2TT, United Kingdom; Department of Metabolism and Systems Science, School of Medical Sciences, College of Medicine and Health, University of Birmingham, Birmingham, B15 2TT, United Kingdom; NIHR Birmingham Biomedical Research Centre, Women's Metabolic Health Theme, University of Birmingham, Birmingham, B15 2TT, United Kingdom; WHO Collaborating Centre for Global Women's Health, University of Birmingham, Birmingham, B15 2TT, United Kingdom; WHO Collaborating Centre for Global Women's Health, University of Birmingham, Birmingham, B15 2TT, United Kingdom; Institute of Life Course and Medical Sciences, Faculty of Health and Life Sciences, University of Liverpool, Liverpool, L7 8TX, United Kingdom; Liverpool Women's Hospital NHS Foundation Trust, Liverpool, Liverpool, L8 7SS, United Kingdom; NIHR Northwest Coast Applied Research Collaboration, University of Liverpool, Liverpool, L7 8TX, United Kingdom; Department of Metabolism and Systems Science, School of Medical Sciences, College of Medicine and Health, University of Birmingham, Birmingham, B15 2TT, United Kingdom; Medical Research Council Laboratory of Medical Sciences, London, W12 0HS, United Kingdom; Institute of Clinical Sciences, Imperial College London, London, SW7 2AZ, United Kingdom; Centre for Endocrinology, Diabetes and Metabolism, Birmingham Health Partners, University of Birmingham, Birmingham, B15 2TT, United Kingdom; Department of Endocrinology and Diabetes, Birmingham Children's Hospital, Birmingham Women's and Children's NHS Foundation Trust, Birmingham, B4 6NH, United Kingdom; Department of Metabolism and Systems Science, School of Medical Sciences, College of Medicine and Health, University of Birmingham, Birmingham, B15 2TT, United Kingdom; NIHR Birmingham Biomedical Research Centre, Women's Metabolic Health Theme, University of Birmingham, Birmingham, B15 2TT, United Kingdom

**Keywords:** androgen excess, puberty, insulin resistance, dyslipidaemia, obesity, pubarche

## Abstract

**Objective:**

Premature adrenarche (PA), characterised by pre-pubertal adrenal androgen excess and hyperandrogenic symptoms, is considered a forerunner of polycystic ovary syndrome, which comes with increased metabolic risk. Here, we aimed to systematically evaluate the evidence on surrogate parameters of metabolic risk in children with PA.

**Methods:**

We searched major databases (1990-March 2025) for studies on PA in children analysing body composition and markers of glucose and lipid metabolism. Two reviewers independently selected studies, collected data, and appraised study quality. Results were standardised, tabulated, and pooled for meta-analysis.

**Results:**

Twenty-five case-control studies reported on 694 children with PA and 567 healthy controls (boys and girls). Height standard deviation score (SDS), weight SDS, and body mass index SDS were significantly higher in PA than in controls. Children with PA also presented with higher fasting insulin (FI) levels than controls (MD: 15.04, 95% CI: 5.27-24.81 pmol/L; *I*^2^ = 91%). These findings persisted after sensitivity analysis for gender and risk of bias assessment. Other markers of metabolic risk, such as fasting glucose, HOMA-IR, mean serum glucose and insulin during the oral glucose tolerance test, and fasting lipids, did not differ between children with PA and controls.

**Conclusions:**

Children with PA are taller, heavier, and have higher FI levels than their healthy peers at presentation. We observed significant heterogeneity of reported outcomes with generally small participant numbers in the included studies. Large-scale studies with comprehensive, unified assessment and long-term follow-up are needed to explore the extent of metabolic dysfunction that may develop over time.

Significance Premature adrenarche (PA) is characterised by androgen excess and may be a forerunner condition of polycystic ovary syndrome (PCOS), the most common endocrine disorder in adult women. Women with PCOS are at higher risk of developing type 2 diabetes, dyslipidaemia, and fatty liver disease. Studies on metabolic risk in PA are limited to small numbers, and results are conflicting. Our systematic review and meta-analysis assessed metabolic risk parameters in children with PA compared with matched controls at presentation. Pooled meta-analysis suggests a higher likelihood of metabolic disturbances in PA characterised by higher fasting insulin in overall taller and heavier children. Consistent data from large studies with unified phenotypic assessment are needed.

## Introduction

Adrenarche reflects the development of the adrenal zona reticularis, the androgen-producing zone of the adrenal cortex, which expresses steroidogenic enzymes capable of producing the key androgen precursor dehydroepiandrosterone (DHEA) and its inactive metabolite DHEA sulphate (DHEAS), the most abundant circulating steroid in young adults.^1,2^ Adrenarche is a physiological process that typically occurs during mid-childhood, accompanied by the appearance of axillary and/or pubic hair, greasy hair, adult-type body odour, and transient growth acceleration.^1-3^ Premature adrenarche (PA) refers to the earlier-than-normal development of adrenarche before the age of 8 years in girls and 9 years in boys.^3^ To define (idiopathic) PA, both clinical signs and elevated adrenal androgens above the age-specific reference range should be present.^4-6^ Still, other rarer causes of early-onset sex steroid excess, such as virilising forms of congenital adrenal hyperplasia (CAH), adrenocortical tumours, and central precocious puberty, must be excluded.^2,7^ The prevalence of PA, although not robustly documented, is reported in up to 8.6% of girls and 1.8% of boys when applying those criteria.^8^

The definition of PA has been subject to debate and was variably used, in particular in earlier studies where researchers only reported on children with clinical signs, predominantly the presence of pubic hair (premature pubarche, PP).^2^ More recent studies consider clinical signs and biochemical evidence of androgen excess to define PA.^1,9^ A widely accepted, “traditional” cut-off to define PA biochemically is based on a DHEAS threshold above 40 mcg/dL (1 µmol/L) found in PA between 40 and 130 mcg/dL.^5^ This chosen threshold is conveniently used as a practical guide rather than a validated diagnostic test.^3^

It is well established that adult women with polycystic ovary syndrome (PCOS), the most common endocrine condition characterised by androgen excess, have higher cardiometabolic risk.^10-12^ The potential link between idiopathic early-onset androgen excess in PA and metabolic risk has been explored over the past 30 years.^1,2^ The first case-control study in the 1990s from a Spanish cohort of girls with PP at presentation reported a higher degree of insulin resistance than matched controls.^13^ Subsequently, more studies reporting on various metabolic risk parameters were published in cohorts in different countries, aiming to assess metabolic risk features, including body composition and glucose and lipid metabolism markers. Still, the results are conflicting (narratively reviewed in^2^). Some studies suggest an association between PA/PP and insulin resistance,^14-18^ but other cardiometabolic risk parameters, such as elevated fasting lipids or high blood pressure, were reported only in some cohorts.^19-21^ Functional ovarian hyperandrogenism or irregular periods were present in only a small number of teenagers with a history of PA/PP, when investigators followed them up into their reproductive years, suggesting that not all girls with PA are at risk of developing PCOS.^22,23^

To date, the impact of idiopathic androgen excess in young children on metabolic dysfunction is unclear, and its potential long-term sequelae are poorly explored. Here, we conducted a systematic review of the literature to pool clinical and biochemical markers associated with metabolic dysfunction.

## Methods

### Study design

This systematic review and meta-analysis of observational studies aimed to determine whether children with PA at presentation have higher metabolic risk parameters compared with healthy peers. The study followed the PRISMA guidelines, and the protocol was registered in PROSPERO (CRD42022336669).

### Eligibility criteria

We included case-control studies published from January 1990 to March 2025. Eligible studies compared children with PA to healthy controls, assessing anthropometric/body composition data and markers of glucose or lipid metabolism. Only studies with at least 5 PA cases were included.

PA was defined as the presence of clinical signs (pubic/axillary hair, body odour, acne, or growth acceleration) before age 8 in girls or 9 in boys, with or without biochemical androgen excess. Studies had to exclude virilising CAH, central precocious puberty, sex steroid-secreting tumours, or pharmacotherapy affecting steroid metabolism. Only studies with age-matched healthy control groups were included.

### Outcomes

We included studies reporting on:

1. Anthropometry/body composition: weight, height, body mass index (BMI) (raw values, *z*-scores, or standard deviation score [SDS]).
2. Glucose metabolism: fasting glucose (FG)/insulin, HOMA-IR, HbA1c, and oral glucose tolerance test (OGTT)-derived glucose/insulin values.
3. Lipid metabolism: fasting total cholesterol (TC), high-density lipoprotein (HDL), low-density lipoprotein (LDL), and triglycerides (TG).

Studies from any clinical setting (population-based, primary, or secondary care) were eligible. Reviews, editorials, conference abstracts, and unpublished studies were excluded.

### Data sources and search strategy

We systematically searched MEDLINE, EMBASE, and the Cochrane Library (1990-March 2025), supplemented by grey literature from Google Scholar. Reference lists of relevant articles were also screened. Controlled vocabulary (MeSH/Emtree) and free-text terms were combined using Boolean operators. The search strategy is detailed in the Appendix. Citations were imported into EndNote 20; duplicates were removed.

### Study selection

Two reviewers (W.B. and I.L.) independently screened titles, abstracts, and full texts to determine eligibility. Disagreements were resolved by consensus or a third reviewer (J.I.).

### Data collection

A standardised Excel sheet was used to extract the following: author, year, country, ethnicity, study design, number and sex of PA cases and controls, and participant age. Data collected included:

1. Anthropometry: height, height SDS/*z*-score, weight, weight SDS/*z*-score, BMI (raw/*z*-score/percentile), waist-hip ratio, weight-for-height.
2. Glucose markers: FG/insulin, OGTT-derived values (2 h, mean), HbA1c, HOMA-IR, FIRI, ISI, insulin response to glucose (IRG).
3. Lipid markers: TG, TC, HDL, LDL, very low-density lipoprotein (VLDL).

### Quality assessment

Study quality was assessed using a modified castle–Ottawa Scale (NOS) for observational studies,^24^ evaluating participant selection, comparability of groups, and outcome measurement. Scores ranged from 1 to 9; studies scoring 6-9 were considered high quality. Two reviewers (W.B. and I.L.) assessed quality independently; disagreements were resolved with a third reviewer (J.I.).

### Statistical analysis

Data were pooled using inverse variance random-effects meta-analysis and reported as mean difference (MD) with 95% confidence intervals (CIs). Where necessary, outcomes were converted to standard international (SI) units. Statistical heterogeneity across the included studies was reported as *I*^2^ statistics. Sensitivity analyses were conducted if heterogeneity exceeded 50%, limiting the analysis to studies with CIs overlapping the pooled estimate. RevMan 5.4 software was used. To prevent duplication, if multiple studies reported the same outcomes from the same cohort, only the one with the larger sample was included for that outcome.

### Subgroup and sensitivity analyses

We performed subgroup analyses by PA definition (comparing studies based on clinical signs only vs those requiring clinical signs plus biochemical androgen excess) and BMI adjustment (comparing BMI-matched/adjusted studies vs unmatched/unadjusted studies).

Sensitivity analyses were also conducted for high-quality studies (NOS 6-9) and studies reporting outcomes exclusively in girls.

## Results

### Study characteristics

**Figure 1.**
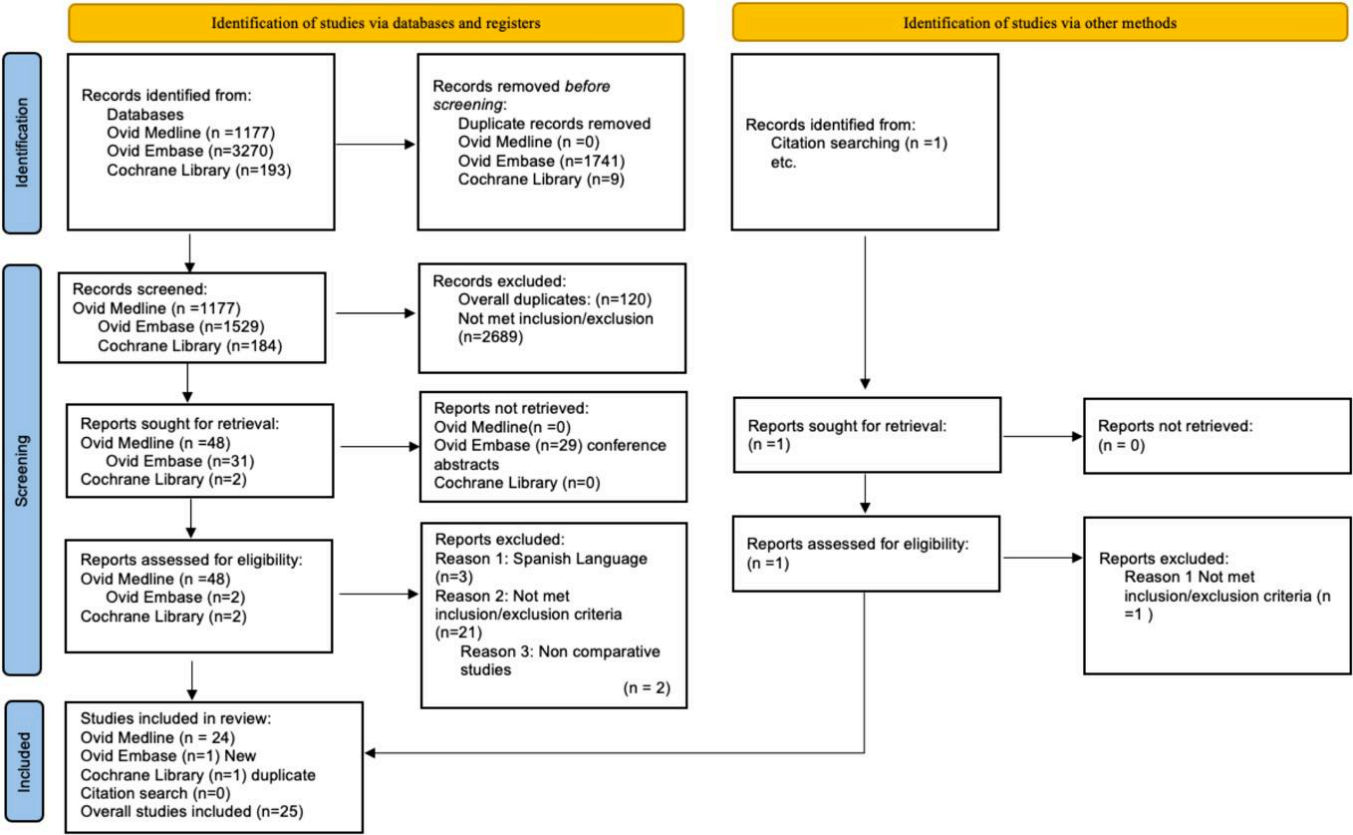
Flow chart of study inclusion for a systematic review and meta-analysis of metabolic risk in premature adrenarche.

A total of 25 case-control studies were included (Figure 1), involving 694 children with PA (616 [88.8%] girls; 78 [11.2%] boys) and 567 healthy controls (482 [85%] girls; 85 [15%] boys) (Table 1). The age at assessment ranged from 3.9 to 10.5 years. Fourteen studies included only girls,^17,18,21,25-35^ 2 only boys,^36,37^ and 9 both sexes.^19,38-45^ Studies were conducted in Turkey (*n* = 8), the USA (*n* = 7), Spain (*n* = 4), Finland (*n* = 3), and 1 study each from Brazil, Greece, and Switzerland. Quality assessment rated 9 studies as high (NOS 6-9) and 16 as low quality (NOS 0-5) (Table 2).

**Table 1.** Characteristics of the included studies; all studies are case-control studies.

Study characteristics				PA diagnosis	Cases			Controls			Outcomes		
No	First author (Year)	Country	Age Mean ± SD Median [IQR] Mean (95% CI) Mean [[SEM]]	Clinical signs (C); clinical signs and biochemical androgen excess (CB)	Total (*n*)	Girls (*n*; %)	Boys (*n*; %)	Total (*n*)	Girls (*n*; %)	Boys (*n*; %)	Anthropometry	Glucose metabolism	Lipid metabolism
1.	Aydin (2022)^25^	Turkey	**PA** 7.71 ± 1.0/**C** 7.54 ± 0.9	**C**	49	49; 100%	—	48	48; 100%	—	BMI SDS, Wt SDS, W:H ratio, Ht SDS	FI, FG, HOMA-IR	TC, LDL, HDL, TG
2.	Topaktas (2021)^38^	Turkey	**PA** 6.7 ± 1.1/**C** 6.5 ± 1.1	**CB**	73	68; 93.1%	5, 6.9%	36	31; 86.1%	5; 13.9%	BMI, BMI SDS, Wt SDS, Ht SDS	FI, FG, HOMA-IR	TC, LDL, HDL, TG
3.	Wise-Oringer (2020)^39^	USA	**PA** 7.4 [6.5-7.8]/**C** 6.3 [3.9-7.0]	**C**	20	15, 75%	5; 25%	11	9; 82%	2; 18%	BMI percentile, BMI *z*-score	FI, FG, HBA1c, HOMA-IR	TG, HDL
4.	Janner (2020)^26^	Switzerland	**PA** 6.7 ± 1.1/**C** 6.5 ± 1.1	**C**	23	23; 100%	—	22	22; 100%	—	BMI, BMI SDS, Wt, Wt SDS, Ht, Ht SDS	/	/
5.	Marakaki (2017)^40^	Greece	**PA** (girls) 7.39 ± 0.95, (boys) 8.95 ± 1.10 **C** (girls) 7.65 ± 1.30, (boys) 8.53 ± 1.26	**C**	82	66; 80.5%	16; 19.5%	63	48; 76.2%	15; 23.8%	BMI SDS, Ht SDS	/	/
6.	Çelik (2017)^19^	Turkey	**PA** 7.61 ± 1.04/**C** 7.46 ± 1.04	**C**	44	36; 81.8%	8; 18.2%	31	21; 67.7%	10; 32.3%	BMI *z*-score, Wt *z*-score	FI, FG, HOMA-IR	TC, LDL, HDL, TG
7.	Williams (2015)^41^	USA	**PA** 7.39 [1.32]/**C** 7.11 [1.14]	**CB**	30	66.7%	33.3%	28	53.6%	46.4%	BMI, BMI *z*-score	FG, HOMA-IR	TAG, HDL
8.	Cebeci (2015)^27^	Turkey	**PA** 7.39 [1.32]/**C** 7.11 [1.14]	**C**	47	47; 100%	—	57	57; 100%	—	BMI, BMI SDS, Wt, Wt SDS, W:H ratio, Ht, Ht SDS	/	/
9.	Utriainen (2009)*^43^	Finland	**PA** 7.5 (± 0.22)/**C** 7.3 (± 0.23)	**CB**	64	54; 84.4%	10; 15.6%	62	52; 83.9%	10; 11%	BMI SDS, Ht SDS	/	/
10.	Leibel (2009)^28^	USA	**PA** 7.98 ± 0.76/**C** 6.73 ± 0.77	**C**	6	100%	—	8	100%	—	BMI *z*-score,	/	/
11	Lappalainen (2009)*^42^	Finland	**PA** 7.98 ± 0.76/**C** 6.73 ± 0.77	**C**	73	63; 86.3%	10; 13.7%	97	79; 81%	18; 18.6%	Ht SDS, Wt for Ht%	HOMA-IR; ISI_comp_,	TC, LDL, HDL, TG
12.	Mathew (2008)^45^	USA	**PA** 7.98 ± 0.76/**C** 6.73 ± 0.77	**C**	10	7; 70%	3; 30%	10	7; 70%	3; 30%	BMI SDS, Wt SDS, W:H ratio, Ht SDS		TC, LDL, HDL, TG
13.	Andiran (2008)^29^	Turkey	**PA** 7.98 ± 0.76/**C** 6.73 ± 0.77	**C**	25	25; 100%	—	20	20; 100%	—	/		TC, LDL, HDL, TG, VLDL
14.	Evliyaoğlu (2007)^30^	Turkey	**PA** 6.93 ± 1.78/**C** 7.55 ± 1.32	**C**	19	19; 100%	—	10	10; 100%	—	BMI SDS, Ht SDS,		TC, LDL, HDL, TG, VLDL-C
15.	Utriainen (2007)*^44^	Finland	**PA** 7.7 (7.5-8.0)/**C** 7.5 (7.3-7.7)	**C**	63	63; 100%	—	80	80; 100%	—	BMI SDS	F. insulin, FG, OGTT: 2 h glucose, MSI; HbA1c; HOMA-IR; ISI	TC, LDL, HDL, TG
16.	Guven (2005)**^21^	Turkey	**PA** 7.1 ± 0.9/**C** 6.9 ± 0.3	**CB**	27	27; 100%	—	13	13; 100%	—	BMI, Wt SDS, Ht SDS	/	/
17.	Guven (2005a)**^31^	Turkey	**PA** 7.6 ± 0.1/**C** 6.9 ± 0.3	**CB**	24	24; 100%	—	13	13; 100%	—	/	FI, FG, HOMA-IR	TC, LDL, HDL, TG, VLDL-C
18.	Teixeira (2004)^32^	Brazil	**PA** 7.3 ± 1.5/**C** 6.4 ± 1.3	**C**	25	25; 100%	—	14	14; 100%	—	BMI, Wt, W:H ratio	FI, FG, F. G/I ratio	TC, LDL, HDL, TG
19.	Ibanez (2003)^33^	Spain	**PA** 8·1 ± [[0.4]]/**C** 8.0 ± [[0.2]]	**CB**	20	20; 100%	—	15	15; 100%	—	BMI, Wt, W:H ratio, Ht	FI	TC, LDL, HDL, TG
20.	Denburg (2002)^36^	USA	**PA** 8.2 ± 0.7/**C** 7.9 ± 0.8	**CB**	11	—	11; 100%	8	—	8; 100%	BMI, BMI *z*-score	FI, proinsulin, FG, HbA1c, Fasting G/I ratio, ISI	TC, LDL, HDL, TG
21.	Silfen (2002)^34^	USA	**PA** 6.9 ± 1.0/**C** 7.6 ± 1.3	**CB**	17	17; 100%	—	9	9; 100%	—	BMI, BMI *z*-score	FI	/
22.	Cizza (2001)^35^	USA	**PA** 7.4 ± [[0.4]]/**C** 7.6 ± [[0.4]]	**C**	7	7; 100%	—	8	8; 100%	—	BMI, Wt, Ht	/	/
23.	Potau (1999)^37^	Spain	**PA** 8.9 ± [[0.2]]/**C** 8.2 ± [[0.5]]	**C**	11	—	11; 100%	9	—	9; 100%	BMI, Wt SDS, Ht SDS	OGTT: MSG, MSI, IRG, SI, M	/
24.	Ibanez (1998)^18^	Spain	**PA** 7.5 ± [[0.2]]/**C** 8.5 ± [[0.3]]	**CB**	21	21; 100%	—	14	14; 100%	—	BMI	OGTT: FIRI, MSG, MSI, IRG, SI, M	TC, LDL, HDL, TG, VLDL, (APO-A, -B, -CII, -CIII, -E)
25.	Ibanez (1997)^17^	Spain	**PA** 7.3 ± [[0.1]]/**C** 8.3 ± [[0.3]]	**C**	32	32; 100%	—	21	21; 100%	—	BMI	OGTT: FIRI, MSG, MSI, IRG, SI, M	/

Abbreviations: Apo-A, apolipoprotein A; Apo-B, apolipoprotein B; Apo-C II, apolipoprotein CII; Apo-CIII, apolipoprotein CIII; ApoE, apolipoprotein E; BMI, body mass index; F, fasting; FG, fasting glucose; FI; fasting insulin; G/I ratio, glucose insulin ratio; HDL, high-density lipoprotein; HOMA-IR, homeostatic model assessment for insulin resistance; Ht, height; IRG, insulin response to glucose; ISI, insulin sensitivity index; LDL, low-density lipoprotein; M, M value (Glucose re-uptake rate in P tissues); MSG; mean serum glucose; MSI, mean serum insulin; PA, premature adrenarche; OGTT, oral glucose tolerance test; SDS, standard deviation score; SI, sensitivity to insulin; TC, total cholesterol; TG, triglycerides; W:H ratio, waist-to-hip ratio; Wt, weight; VLDL, very low-density lipoprotein.

Cohorts marked with (*) and (**) describe studies from the same cohort.

**Table 2.** Adapted Newcastle–Ottawa scale assessment for the included studies.

Study	Selection					Comparability	Outcomes	Overall score
First author (Year)	Representativeness of PA case	Selection of the control group	Sample size	Non-response rate	Ascertainment of the exposure	Adjustment of confounding factors	Statistical test	
Ibanez (1997)^17^	0	0	0	0	1	1	1	3
Ibanez (1998)^18^	0	0	0	0	1	1	1	3
Potau (1999)^23^	0	0	0	0	1	1	1	3
Cizza (2001)^35^	1	1	0	0	1	1	1	5
Denburg (2002)^36^	0	0	0	0	1	2	1	4
Silfen (2002)^34^	0	0	0	0	1	2	1	4
Ibanez (2003)^46^	0	0	0	0	1	2	1	4
Teixeira (2004)^32^	0	0	0	0	1	0	0	1
Guven (2005a)^21^	1	1	0	0	1	1	1	5
Guven (2005)^31^	1	1	0	0	1	1	1	5
Utriainen (2007)^44^	1	1	0	1	2	1	1	6
Evliyaoglu (2007)^30^	0	1	0	0	1	1	1	4
Andiran (2008)^29^	0	1	0	0	2	1	1	5
Mathew (2008)^45^	1	1	0	0	1	2	1	6
Lappalainen (2009)^42^	1	1	0	0	2	1	1	6
Leibel (2009)^28^	1	0	0	0	1	2	1	5
Utriainen (2009)^4^	1	1	0	0	2	1	1	6
Cebeci (2015)^27^	1	1	0	0	1	1	1	5
Williams (2015)^41^	1	0	0	0	2	2	1	6
Celik (2017)^19^	1	1	0	0	1	1	1	5
Marakaki (2017)^40^	1	0	0	0	2	2	1	6
Janner (2020)^26^	1	1	0	0	1	1	1	5
Wise-Oringer (2020)^39^	1	1	0	0	2	2	1	7
Topaktas (2021)^38^	1	0	1	0	2	1	1	6
Aydin (2022)^25^	1	0	0	0	2	2	1	6

Abbreviation: PA, premature adrenarche.

Overall score 6-9: high quality (grey shaded); overall score 0-5: low quality.

### Anthropometric outcomes

#### Height SDS

**Figure 2.**
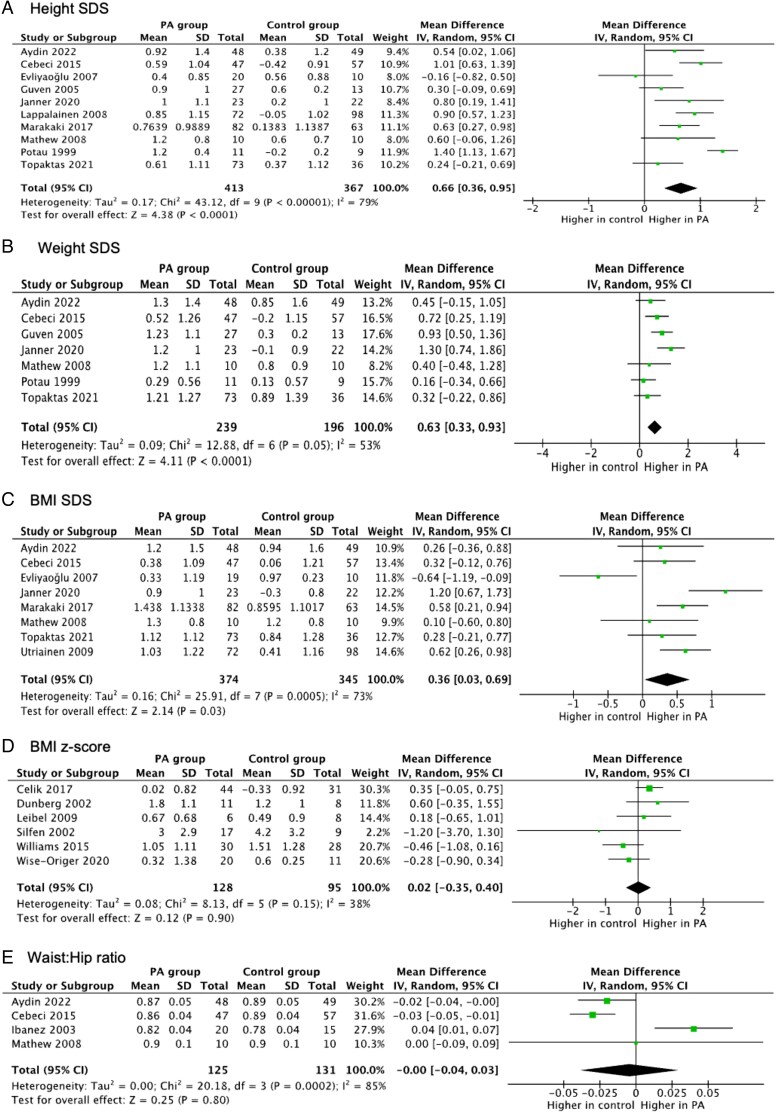
Forest plots for meta-analysis of anthropometric outcomes in children with premature adrenarche (PA) compared with children without PA. (A) Forest plot for height standard deviation score (SDS); (B) forest plot for weight SDS; (C) forest plot for BMI SDS; (D) forest plot for BMI *z*-score; and (E) forest plot for waist:hip ratio.

Ten studies reported on height SDS (35-37, 40, 41, 47, 48, 50, 52, 55), with 6 showing significantly higher values in PA children (35-37, 47, 50, 52). The pooled MD was 0.66 (95% CI: 0.36-0.95; *I*² = 79%) (Figure 2A). Sensitivity analysis, excluding the outlier study,^37^ reduced heterogeneity (*I*² = 55%, MD: 0.57). Subgroup analysis indicated greater height SDS in PA compared with controls diagnosed by clinical signs alone (*P* = .03, Table S2). In high-quality studies,^25,38,40,42,45^ the difference remained (MD: 0.61; 95% CI: 0.38-0.84; *I*² = 28%). Among girls-only studies,^25-27,30,31^ the difference persisted with reduced heterogeneity (*I*² = 67%; Table S5).

#### Weight SDS

Seven studies examined weight SDS,^25-27,31,37,38,45^ with 3 showing significantly higher values in PA compared with controls.^26,27,31^ Meta-analysis showed higher weight SDS in PA compared with controls (MD: 0.63; 95% CI: 0.33-0.93; *I*² = 53%) (Figure 2B). Subgroup analyses by PA definition showed no significant difference (*P* = .86). Weight SDS remained significantly higher in PA in high-quality^25,26,38,45^ and girls-only studies^25-27,31^ (MDs: 0.64 and 0.86, respectively) (Tables S4 and S5).

#### BMI SDS

Eight studies assessed BMI SDS,^25-27,30,38,40,43,45^ with 3 reporting significantly higher values in PA.^26,40,43^ Overall, BMI SDS was higher in PA (MD: 0.36; 95% CI: 0.03-0.69; *I*² = 73%) (Figure 2C). Exclusion of the outlier study^30^ lowered *I*² to 43% (MD: 0.51; 95% CI: 0.27-0.75). Studies for clinical-only^25,38^ and clinical + biochemical^26,27,30,40,43,45^ PA showed elevated BMI SDS (*P* = .7) in the subgroup analysis (Table S2). In high-quality studies,^25,38,40,43,45^ BMI SDS remained higher (MD: 0.46; 95% CI: 0.26-0.67; *I*² = 0%). In girls-only studies,^25-27,30^ the trend did not reach significance (Table S5).

#### BMI *z*-score

Six studies^19,28,34,36,39,41^ showed no significant difference in BMI *z*-score between PA and controls (MD: 0.02; 95% CI: −0.35 to 0.4; *I*² = 38%) (Figure 2D). Subgroup and sensitivity analyses were not feasible due to small sample sizes.

#### Waist-to-hip ratio

Four studies^25,27,33,46^ assessed waist-to-hip ratio (WHR). One found higher WHR in PA,^33^ another in controls.^27^ Meta-analysis showed no overall difference (MD: 0.0; 95% CI: −0.04 to 0.03; *I*² = 85%) (Figure 2E).

### Glucose metabolism

#### Fasting insulin

**Figure 3.**
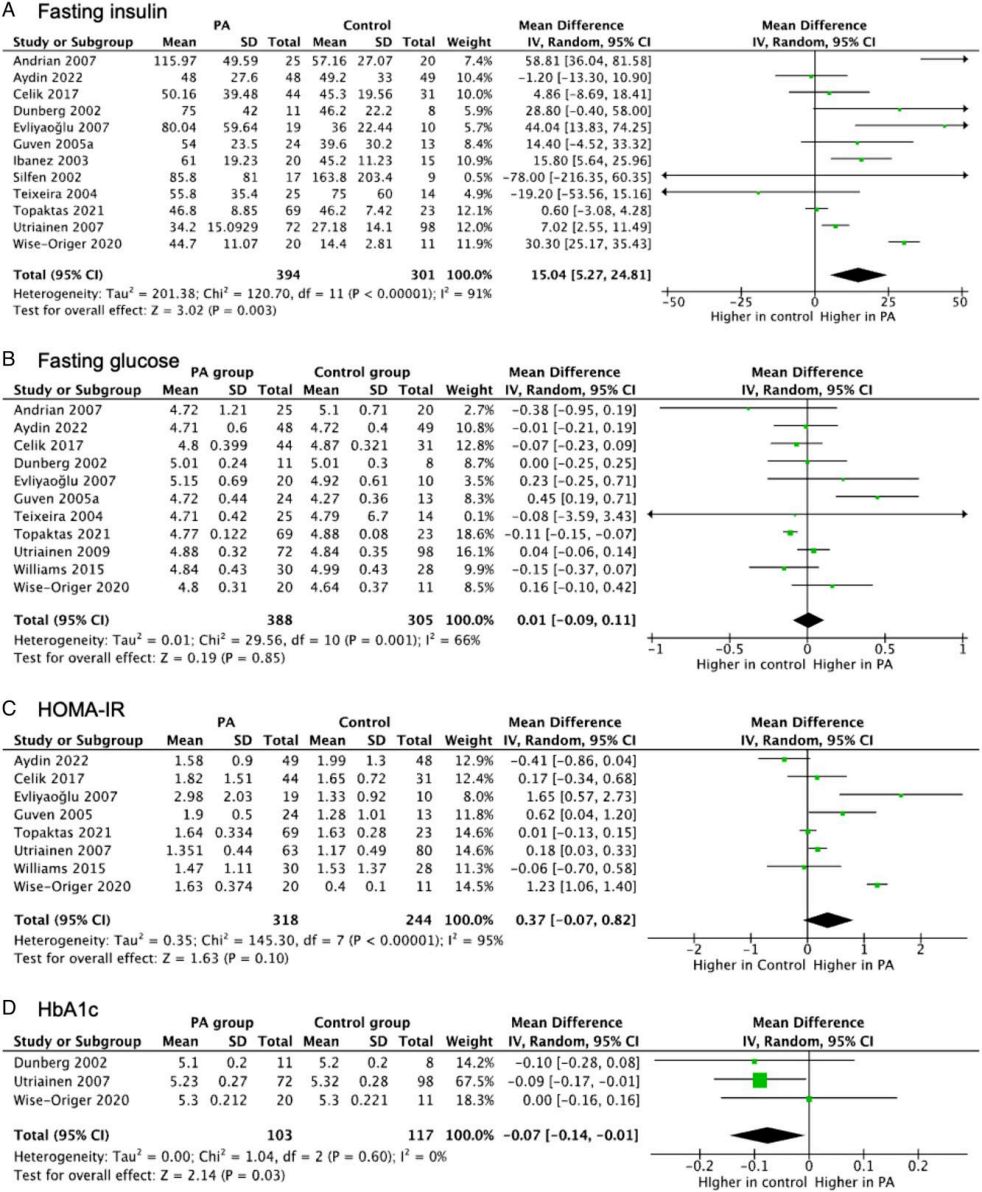
Forest plots for meta-analysis of glucose metabolism outcomes in children with premature adrenarche (PA) compared with children without PA. (A) Forest plot for fasting insulin; (B) forest plot for fasting glucose; (C) forest plot for HOMA-IR; and (D) forest plot for HbA1c.

Twelve studies^19,21,25,29,30,32-34,36,38,39,44^ assessed fasting insulin (FI); 5 showed significantly higher levels in PA.^25,30,33,39,44^ Overall, FI was significantly higher in PA (MD: 15.04; 95% CI: 5.27-24.81; *I*² = 91%) (Figure 3A). There was no significant difference between PA and controls in subgroup analyses for clinical-only vs clinical-biochemical (*P* = .15) (Table S2) and in studies adjusting vs non-adjusting for BMI (*P* = .85; Table S3). Sensitivity analyses, including high-quality^25,38,39,44^ and girls-only studies,^21,25,29,30,32-34,39^ confirmed no difference in FIFI.

#### Fasting glucose

Eleven studies^19,21,25,29,30,32,36,38,39,41,43^ were analysed. One study each reported higher glucose in PA^21^ and controls.^38^ No overall difference was found (MD: 0.01; 95% CI: −0.09 to 0.11; *I*² = 66%) (Figure 3B). Excluding the outlier study^21^ reduced *I*² to 38%. Subgroup and sensitivity analyses did not change the outcomes (Tables S2-S5).

#### HOMA-IR

Eight studies^19,25,30,31,38,41,44^ evaluated HOMA-IR. Three reported significantly higher values in PA,^30,31,39^ but the pooled estimate was non-significant (MD: 0.37; 95% CI: −0.07 to 0.82; *I*² = 95%) (Figure 3C). Sensitivity analysis excluding the outlier study^39^ nullified the difference but reduced *I*^2^ to 68%. No subgroup differences were observed (Tables S2-S5).

#### HbA1c

**Figure 4.**
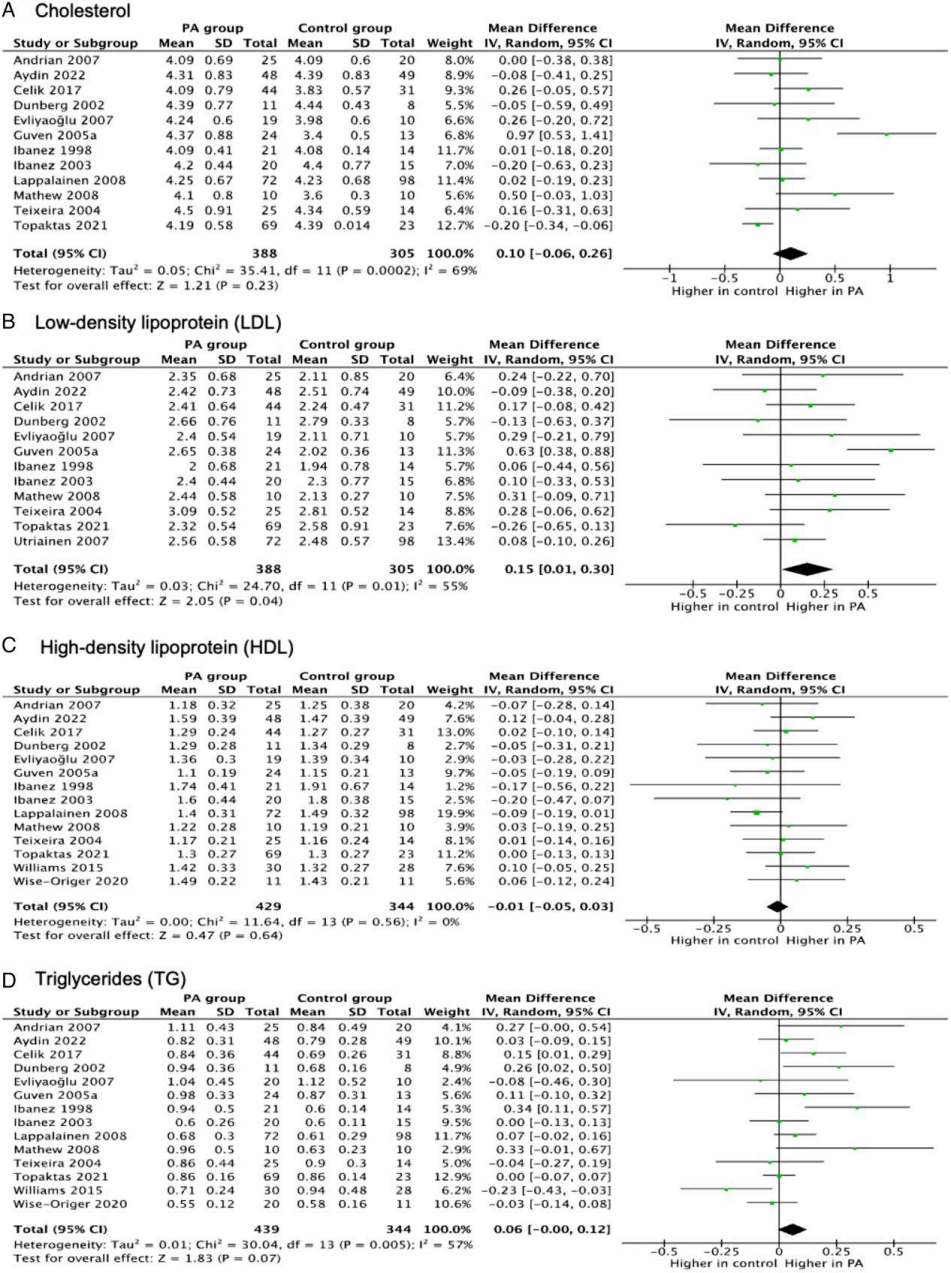
Forest plots for meta-analysis of lipid metabolism outcomes in children with premature adrenarche (PA) compared with children without PA. (A) Forest plot for total cholesterol; (B) forest plot for low-density lipoprotein (LDL); (C) forest plot for high-density lipoprotein (HDL); and (D) forest plot for triglycerides.

Three studies showed lower HbA1c in PA,^36,39,44^ one in controls.^44^ Overall, HbA1c was higher in controls than PA (MD: −0.07; 95% CI: −0.14 to −0.01; *I*² = 0%) (Figure 4D). Further analyses were not meaningful due to the small number of included subjects.

#### Fasting glucose/insulin ratio

Four studies^29,30,32,36^ reported a lower fasting glucose/insulin ratio (FGIR) in PA (MD: −7.5, 95% CI: −11.34 to −3.65; *I*^2^ = 0%). Subgroup and sensitivity analyses were not performed due to the small number of included studies.

#### Mean serum glucose and mean serum insulin during OGTT

Three studies each assessed mean serum glucose (MSG) and mean serum insulin (MSI).^17,18,37^ Mean serum glucose was not different (MD: 0.12; 95% CI: −0.41 to 0.66; *I*² = 44%) and MSI was significantly higher in PA (MD: 0.78; 95% CI: 0.14-1.43; *I*² = 60%). Further analyses were not meaningful due to the small number of included subjects.

### Lipid metabolism

#### Fasting total cholesterol

Twelve studies^18,19,25,29-33,36,38,42,45^ assessed TC. One study reported significantly higher levels in PA.^21^ No overall difference was found (MD: 0.1; 95% CI: −0.06 to 0.26; *I*² = 69%) (Figure 4A). Sensitivity and subgroup analyses did not change the outcomes (Tables S2-S5).

#### Low-density lipoprotein

Twelve studies^18,19,21,25,29,30,32,33,36,38,44,45^ reported on LDL. One found significantly higher levels in PA.^21^ The pooled estimate revealed higher LDL levels in PA than in controls (MD: 0.15; 95% CI: 0.01-0.30; *I*² = 55%) (Figure 4B). When removing the outlier study,^21^ there was no difference (*I*² = 0%). Higher LDL was observed in studies that did not adjust for BMI. Subgroup analysis showed higher LDL in PA compared with controls in studies that did not adjust for BMI (MD: 0.25; 95% CI: 0.03-0.47; *I*^2^ = 63%) (Table S3). Further subgroup and sensitivity analyses did not significantly change the outcomes (Tables S2, S4, and S5).

#### High-density lipoprotein

In 14 studies,^18,19,21,29,30,32,33,36,38,39,41,42,45,47^ HDL was not significantly different between PA and controls (Figure 4C). Subgroup analysis by PA definition and BMI adjustment showed no difference. However, higher HDL in PA was noted in BMI-matched/adjusted studies (*P* = .01) (Tables S2-S5).

#### Fasting triglycerides

Fourteen studies^18,19,21,25,29,30,32,33,36,38,39,41,42,45^ assessed TG; 3 showed higher levels in PA,^18,19,36^ 1 in controls.^41^ Pooled MD showed a non-significant trend towards high TG levels in PA (0.06; 95% CI: −0.00 to 0.12; *I*² = 57%) (Figure 4D). Removing outliers^18,41^ did not affect the outcome. Subgroup analyses found no differences (Tables S2-S5).

### Other outcomes not qualified for meta-analysis

Additional outcomes, such as BMI percentiles,^39^ weight-for-height percentages,^42^ and OGTT indices (QUICKI, insulin sensitivity index, 2 h insulin/glucose),^17,18,33,34,36,37,42-44,48^ showed no significant differences between groups, except in 1 Finnish cohort where post-OGTT insulin/glucose was higher in PA girls.^44^ Apoprotein A was assessed in 3 studies^18,21,29^ and was only significantly higher in PA in 1 study.^21^

## Discussion

This is the first systematic review with meta-analyses on metabolic risk parameters in children with PA at presentation. We have previously narratively reviewed the link between PA and dysfunction and reported conflicting results.^2^ Herein, we pooled various metabolic outcomes for meta-analysis derived from case-control studies focusing on elements of the metabolic syndrome, including body composition and surrogate markers of glucose and lipid metabolism, aiming to obtain a more detailed assessment of metabolic risk factors in PA at presentation. We only included studies reporting on children with PA and did not include studies exploring associations between androgen levels and metabolic dysfunction to maintain a clinical focus. Our analysis confirms that children with PA are taller and heavier; they show subtle metabolic disturbances such as higher FI levels with trends towards higher HOMA-IR and higher LDL cholesterol with trends towards higher TG and TC.

The finding that children with PA are taller and heavier than their peers is consistent in most case-control studies included in this review, often a presenting complaint to seek healthcare attention.^1,44,49^ Adrenal androgens promote growth acceleration by stimulating epiphyseal growth^50^ and osteoblast proliferation,^51^ resulting in bone age advancement and (transient) tall stature. Bone age is significantly advanced in PA and often associated with excess weight,^40,52^ but in most cases, within 2 standard deviations of normal.^1,49,53^ Adult height is not compromised in PA, although pubertal development shifted to a slightly younger age.^54,55^

Higher childhood body weight is associated with higher adrenal androgens,^56^ making it challenging to dissect the individual contribution of body weight or androgen excess in PA on growth and metabolic dysfunction. The mechanisms underlying the well-documented association of excess adipose and excess (adrenal) androgens include higher activity of the hypothalamic–pituitary–adrenal axis,^57^ stimulatory effects of leptin on adrenal androgen biosynthesis^58^ and hyperinsulinism.^59^ Hyperinsulinism has also been associated with adiposity in girls with PA.^60^ A similar complex interplay is part of the pathogenesis of PCOS, where hyperandrogenism exacerbates (central) adiposity, increasing insulin resistance and further promoting visceral fat deposition.^61-63^ As a crude marker, BMI has limitations as it cannot distinguish between fat and muscle mass. Central “android” fat has a stronger association with cardiometabolic risk in longitudinal studies of obese children^64^ and women with PCOS.^65^ Waist circumference, WHR, or waist-to-length ratios are better indices in assessing metabolic risk factors, superior to BMI,^64^ and were reported in 4 studies included in this review. However, only 1 study reported a higher WHR in the PA group,^33^ but in 2 studies, WHR was higher in controls.^25,27^ Future studies in PA should employ more detailed methods to assess body composition, such as dual-energy X-ray absorptiometry (DXA), the “gold standard” for assessing compartmental fat (and lean mass) distribution.^66^

A range of surrogate markers of glucose metabolism were employed in the included studies, the most common being FG and FI. In our meta-analysis, only FI levels were significantly higher in the PA group, and a trend towards higher HOMA-IR in PA was observed. Fasting insulin is considered the most practical approach in assessing insulin resistance. It is also considered the first step of evolving dysglycaemia before FG or even HbA1c changes occur.^67^ However, we observed heterogeneity in our meta-analysis (*I*^2^ = 91%); 7 (out of 12) studies did not observe significantly higher insulin levels in their PA groups. The euglycaemic hyperinsulinaemic clamp is considered the gold standard in assessing insulin sensitivity in children, an extremely invasive and resourceful procedure; however, there is no clear standard for how insulin resistance is best assessed in children based on less-invasive surrogate markers.^68^ Fasting insulin does not always correlate well with clamp data^69^ and relies on accurate assays.^68^ The outcome data analysed in our meta-analysis was reported over 2 decades with different insulin assays employed, likely a key contributor to heterogeneity. Nevertheless, fasting hyperinsulinaemia is considered the first step towards insulin resistance before (fasting) glucose levels are affected,^67^ also reflected in our meta-analysis. The meta-analysis of studies reporting on HOMA-IR does not show a significant signal in PA children. Our subgroup analysis does not show any significant differences in BMI-matched and unmatched studies, indicating that dissecting the contribution of body weight/obesity in PA on glucose metabolism markers is challenging.

Ethnic differences in beta-cell adaptation in children and adolescents have been reported.^70^ They may also contribute to the observed heterogeneity since most studies were conducted in Turkish and Spanish children, harbouring a higher cardiometabolic risk profile.^70,71^ Indeed, consistent signals of insulin resistance, based on insulin levels during an OGTT and other indices, were reported in 2 larger cohorts of Catalan PA girls, which persisted during puberty.^16-18^ A milder insulin resistance signature was reported in a Finnish cohort of PA girls,^44^ and not all of these girls developed or maintained insulin resistance post-puberty.^15,72^

Our analysis suggests trends towards impaired lipid metabolism, including higher TG, TC, and LDL with HDL. Again, stark heterogeneity was observed. Guven^21^ reported significantly higher TC and LDL in a small sample of Turkish PA girls, whereas Ibanez^18^ and Denberg^36^ observed higher TG in Catalonian girls and an ethnically mixed group of PA boys, respectively. Such differences could not be replicated in some White cohorts,^39,44^ suggesting that ethnicity may contribute to an unfavourable metabolic risk profile. Our attempt to analyse subgroup differences for BMI-matched vs BMI-unmatched studies, however, did reveal subgroup differences for HDL cholesterol (higher in PA with BMI-matched controls) and trends towards higher total/LDL cholesterol and TG in PA when not BMI-matched to controls, suggesting that body weight/obesity contributes to dyslipidaemia in PA.

Indeed, obesity is the leading cause of secondary paediatric dyslipidaemia, amenable to weight loss/lifestyle modifications as the first line of treatment.^73^ Dyslipidaemia, in conjunction with insulin resistance and obesity, is frequently observed in women with PCOS, even at a younger age, and is thought to play a role in its development.^74^ Equally, androgens have been identified as independent drivers of dyslipidaemia and cardiovascular dysregulation in PCOS, but also in transgender men.^75^ With the current evidence, due to the paucity of appropriately powered long-term follow-up studies, future cardiometabolic risk has not been identified in children with PA.^15^

The observed degree of heterogeneity may also derive from the inclusion criteria applied. Historically, there has been a lack of consensus to define or diagnose PA; the emergence of pubic hair, ie, PP, has been used synonymously with PA.^13,16-18^ Whilst the development of pubic hair is a clinical manifestation of adrenarche, other clinical symptoms, such as body odour or greasy hair, would be neglected. In addition, the biochemical confirmation of increased androgen levels would be missed. Some investigators have defined PA strictly as elevated adrenal androgens without taking into account the clinical manifestation.^5^ Currently, the widely accepted definition of PA is the presence of clinical signs *and* elevated androgen levels above the age- and sex-specific reference range.^1-3^ From all studies included, only 8/25 recruited PA subjects based on clinical *and* biochemical grounds.^18,21,31,33,34,36,38,41^ This has prompted us to perform a subgroup analysis comparing children with PA included based on clinical *and* biochemical androgen excess vs subjects included based on clinical grounds only. Surprisingly, we observed significant subgroup differences for height SDS (higher in the “clinical PA” subgroup) and higher mean differences for FI and LDL cholesterol in the subgroup of children included based on clinical symptoms, but no differences in weight/BMI or age. It was not possible for us to determine which subjects from the “clinical PA” subgroup had normal adrenal androgen (DHEAS) levels or what the exact clinical presenting signs were since 1 study suggests that girls presenting with PP had more extenuated metabolic changes than girls presenting with body odour or greasy hair (non-PP).^72^

PA frequently manifests in girls with a prevalence of about 9%-20% in girls and 2%-10% in boys, pending on which criteria are used.^1,8,9^ In the studies included in this review, 14 studies reported in girls-only, 2 studies in boys, and 9 presented a mixed cohort with an overall representation of girls at 85% of the cumulative study subjects. Due to the limited number of studies reporting on boys, we could not perform a subgroup analysis for gender. We attempted a sensitivity analysis excluding boys-only and mixed studies. However, this has not changed any of the outcomes.

DHEAS is considered the key classic adrenal androgen and a marker of adrenarche.^1,2^ Recent work has highlighted that adrenally derived 11-oxygenated androgens, a long-time known, previously neglected but re-discovered androgen class due to their high androgenic capability,^76,77^ are the predominant circulating androgens in (premature) adrenarche.^39,78^ However, how they correlate to metabolic risk parameters in PA (and PCOS) is still unclear and should be addressed in future studies.

The findings of the present review should be interpreted in the context of its limitations. Although we were able to pool some key parameters as components of metabolic dysfunction statistically, the summary measures were widely heterogeneous, which is likely a combination of different inclusion criteria used, variation of biochemical assays employed over a considerable time period (28 years), and variation in recruiting/matching healthy controls to the PA groups. Nevertheless, our data offer, for the first time, a systematic pooled analysis of case-control cohorts reporting on metabolic dysfunction in PA at presentation, suggesting early metabolic changes that warrant further exploration.

## Summary and conclusions

In summary, our present review suggests that children with PA at presentation show early signs of metabolic dysfunction, in addition to tall stature and heavier body weight for height. We observed significant heterogeneity. The use of different PA definitions, ethnic variation, and the contributing impact of obesity, in addition to androgen excess, likely causes this bias. Future studies should address this by employing unified inclusion criteria, higher participant numbers with appropriately matched controls, and long-term follow-up to explore which children are at higher risk of developing metabolic disease in adolescence and beyond.

## Supplementary Material

lvaf167_Supplementary_Data
