# Supplementary material for: Premature adrenarche and metabolic risk: a systematic review and meta-analysis
Source: Eur J Endocrinol. 2025 Aug 30;193(3):S1–S14. doi: 10.1093/ejendo/lvaf167 (PMC12397577; doi:10.1093/ejendo/lvaf167)
Supplement: lvaf167_Supplementary_Data [file lvaf167_supplementary_data.docx]

SUPPLEMENTAL APPENDIX

Premature Adrenarche and Metabolic Risk: A Systematic Review and Meta-analysis

Wogud Ben Said^1,2,3,4^, Ioannis G Lempesis^1,3^, Silvia Fernandez-Garcia^3,4,5^, Shakila Thangaratinam^5,6,7,8^, Wiebke Arl^3,9,10^ and Jan Idkowiak^1,2,3,4^

1. Centre for Endocrinology, Diabetes and Metabolism, Birmingham Health Partners, University of Birmingham, United Kingdom
2. Department of Endocrinology and Diabetes, Birmingham Children’s Hospital, Birmingham Women’s and Children’s NHS Foundation Trust, United Kingdom
3. Department of Metabolism and Systems Science, School of Medical Sciences, College of Medicine and Health, University of Birmingham, United Kingdom
4. NIHR Birmingham Biomedical Research Centre, Women’s Metabolic Health Theme, University of Birmingham, United Kingdom
5. WHO Collaborating Centre for Global Women’s Health, University of Birmingham, Birmingham, United Kingdom
6. Institute of Life Course and Medical Sciences, Faculty of Health and Life Sciences, University of Liverpool, United Kingdom
7. Liverpool Women’s Hospital NHS Foundation Trust, Liverpool, United Kingdom
8. NIHR Northwest Coast Applied Research Collaboration, University of Liverpool, Liverpool, United Kingdom
9. Medical Research Council Laboratory of Medical Sciences, London, United Kingdom
10. Institute of Clinical Sciences, Imperial College London, London, United Kingdom

**Supplementary Table 1: Search strategy**

| **Medline** | **Embase** | **Cochrane** |
| --- | --- | --- |
| exp Adrenarche/. | exp Adrenarche/. | Adrenarche |
| Adrenarche/ or premature adrenarche.mp. | Adrenarche/ or premature adrenarche.mp. | MeSH descriptor: [Adrenarche] explode all trees |
| pubarche.mp. | pubarche.mp. | (premature NEXT (adrenarche* or pubarche* or axiilarche*)):ti,ab,kw OR (adrenal hyperandrogegism):ti,ab,kw OR (precocious next (adrenarche* or pubarche* or axiilarche*)):ti,ab,kw OR (androgen excess):ti,ab,kw |
| exp Puberty, Precocious/ or premature pubarche.mp. | exp Puberty, Precocious/ or premature pubarche.mp. | #1 OR #2 OR #3 |
| axillarche.mp. | axillarche.mp. | MeSH descriptor: [Glycated Hemoglobin A] explode all trees |
| body odour.mp. | body odour.mp. | ("hba1c" OR "glycated hemoglobin A"):ti,ab,kw |
| adrenal hyperandrogenism.mp. | adrenal hyperandrogenism.mp. | (fasting next (glucose or insulin)):ti,ab,kw |
| androgen excess.mp. | androgen excess.mp. | MeSH descriptor: [Metabolic Syndrome] explode all trees |
| 1 or 2 or 3 or 4 or 5 or 6 or 7 or 8 | 1 or 2 or 3 or 4 or 5 or 6 or 7 or 8 | (metabolic next (syndrome or dysfunction or risk*)):ti,ab,kw |
| hba1c.mp. or exp Glycated Hemoglobin A/ | hba1c.mp. or exp Glycated Hemoglobin A/ | MeSH descriptor: [Insulin Resistance] explode all trees |
| insulin sensitivity.mp. or exp Insulin Resistance/ | insulin sensitivity.mp. or exp Insulin Resistance/ | (insulin next (sensitivity or resistance or insensitivity)):ti,ab,kw |
| exp Obesity/ or fasting insulin.mp. | exp Obesity/ or fasting insulin.mp. | MeSH descriptor: [cardiovascular diseases] explode all trees |
| fasting glucose.mp. | fasting glucose.mp. | (cardiovascular next (disease* or disorder*)):ti,ab,kw OR (heart next (disease* or disorder*)):ti,ab,kw OR ((cardiac or vascular) next disease*):ti,ab,kw |
| metabolic syndrome.mp. or exp Metabolic Syndrome/ | metabolic syndrome.mp. or exp Metabolic Syndrome/ | MeSH descriptor: [Glucose Tolerance Test] explode all trees |
| metabolic dysfunction.mp. | metabolic dysfunction.mp. | (homa-ir):ti,ab,kw |
| metabolic risk.mp. | metabolic risk.mp. | MeSH descriptor: [Lipids] explode all trees |
| cardiovascular risk.mp. or exp Heart Disease Risk Factors/ | cardiovascular risk.mp. or exp Heart Disease Risk Factors/ | (lipid* or triglyceride* or cholesterol):ti,ab,kw |
| oral glucose tolerance test.mp. or exp Glucose Tolerance Test/ | oral glucose tolerance test.mp. or exp Glucose Tolerance Test/ | MeSH descriptor: [Cholesterol] explode all trees |
| ogtt.mp. | ogtt.mp. | MeSH descriptor: [Body Composition] explode all trees |
| homa-ir.mp. | homa-ir.mp. | MeSH descriptor: [Adipose Tissue] this term only |
| lipid$.mp. or exp Lipids/ | lipid$.mp. or exp Lipids/ | MeSH descriptor: [Body Constitution] explode all trees |
| triglyceride$.mp. or exp Triglycerides/ | triglyceride$.mp. or exp Triglycerides/ | (body next (composition or fat or mass index)):ti,ab,kw |
| exp Cholesterol/ or Cholesterol.mp. | exp Cholesterol/ or Cholesterol.mp. | (body next (composition or fat or mass index)):ti,ab,kw |
| body composition.mp. or exp Body Composition/ | body composition.mp. or exp Body Composition/ | #4 and #23 |
| body fat.mp. or exp Adipose Tissue/ | body fat.mp. or exp Adipose Tissue/ |  |
| bmi.mp. or exp Body Mass Index/ | bmi.mp. or exp Body Mass Index/ |  |
| 10 or 11 or 12 or 13 or 14 or 15 or 16 or 17 or 18 or 19 or 20 or 21 or 22 or 23 or 24 or 25 or 26 or 27 | 10 or 11 or 12 or 13 or 14 or 15 or 16 or 17 or 18 or 19 or 20 or 21 or 22 or 23 or 24 or 25 or 26 or 27 |  |
| 9 and 28 | 9 and 27 |  |
| limit 29 to (human and yr="1990 -Current") | limit 28 to (human yr="1990 -July 2023") |  |
|  | Limit 29 to “remove Medline records” |  |

Supplementary Table 2: Subgroup analysis by PA definition

| **Outcomes** | **Clinical and biochemical PA** | | | | **Clinical PA only** | | | | **Subgroup difference** | | |
| --- | --- | --- | --- | --- | --- | --- | --- | --- | --- | --- | --- |
|  | **No of studies** | **PA**  **(N)** | **Control**  **(N)** | **Mean difference (95%CI)** | **No of studies** | **PA**  **(N)** | **Control**  **(N)** | **Mean difference (95%CI)** | **I^2^** | **P value** |  |
| **Anthropometry** |  |  |  |  |  |  |  |  |  |  |  |
| Height SDS | 3 | 148 | 98 | 0.34 (0.08, 0.59) | 7 | 265 | 269 | 0.8 (0.46, 1.13) | 78.1% | **0.03** |  |
| Weight SDS | 3 | 148 | 98 | 0.6 (0.21, 1.00) | 4 | 91 | 98 | 0.66 (0.15, 1.17) | 0% | 0.86 |  |
| BMI SDS | 2 | 121 | 85 | 0.27 (-0.11, 0.66) | 6 | 253 | 260 | 0.38 (-0.05, 0.81) | 0% | 0.70 |  |
| BMI Z-score | 4 | 64 | 53 | -0.04 (-0.63, 0.54) | 2 | 64 | 42 | 0.08 (-0.53, 0.69) | 0% | 0.77 |  |
| **Markers of glucose  metabolism** |  |  |  |  |  |  |  |  |  |  |  |
| Fasting insulin | 6 | 189 | 117 | 7.65 (-1.61, 16.9) | 6 | 205 | 184 | 21.1 (5.38, 36.82) | 52.1% | 0.15 |  |
| Fasting glucose | 5 | 182 | 121 | 0.01 (-0.16, 0.18) | 6 | 206 | 184 | 0.02 (-0.06, 0.10) | 0% | 0.94 |  |
| HOMA-IR | 5 | 231 | 190 | 0.21 (-0.35, 0.77) | 3 | 87 | 54 | 0.67 (-0.01, 1.36) | 3.6% | 0.31 |  |
| **Markers of lipid metabolism** |  |  |  |  |  |  |  |  |  |  |  |
| Cholesterol | 6 | 193 | 122 | 0.05 (-0.22, 0.31) | 6 | 195 | 183 | 0.13 (-0.01, 0.26) | 0% | 0.59 |  |
| Triglycerides | 7 | 265 | 220 | 0.07 (0.00, 0.14) | 7 | 174 | 124 | 0.04(-0.10, 0.17) | 0% | 0.65 |  |
| LDL | 6 | 193 | 122 | 0.07 (-0.25, 0.39) | 6 | 195 | 183 | 0.17 (0.05, 0.28) | 0% | 0.58 |  |
| HDL | 8 | 295 | 248 | -0.02 (-0.9, 0.05) | 6 | 134 | 96 | 0.01 (-0.06, 0.08) | 0% | 0.55 |  |

**Supplementary Table 3:** Subgroup analysis on the effect of BMI

| **Outcomes** | **adjusted/matched for BMI** | | | | **Not adjusted/ matched for BMI** | | | | **Subgroup difference** | | |
| --- | --- | --- | --- | --- | --- | --- | --- | --- | --- | --- | --- |
|  | **No of studies** | **PA**  **(N)** | **Control**  **(N)** | **Mean difference (95%CI)** | **No of studies** | **PA**  **(N)** | **Control**  **(N)** | **Mean difference (95%CI)** | **I^2^** | **P value** |  |
| **Markers of glucose  metabolism** |  |  |  |  |  |  |  |  |  |  |  |
| Fasting insulin | 7 | 228 | 141 | 14.35 (-1.66-30.35) | 5 | 166 | 160 | 16.47 (1.68-31.26) | 0% | 0.85 |  |
| Fasting glucose | 7 | 242 | 160 | -0.07 (-0.14-0.00) | 4 | 146 | 145 | 0.10 (-0.24-0.44) | 0% | 0.34 |  |
| HOMA-IR | 8 | 231 | 151 | 0.39 (-0.27-1.05) | 2 | 87 | 93 | 0.31 (-0.08-0.69) | 0% | 0.84 |  |
| **Markers of lipid metabolism** |  |  |  |  |  |  |  |  |  |  |  |
| Cholesterol | 6 | 201 | 131 | 0.07 (-0.16-0.31) | 6 | 187 | 174 | 0.13 (-0.11-0.38) | 0% | 0.72 |  |
| Triglycerides | 8 | 252 | 170 | 0.03 (-0.05-0.12) | 6 | 187 | 174 | 0.10 (0.00-0.20) | 0% | 0.32 |  |
| LDL | 6 | 201 | 131 | 0.05 (-0.13-0.23) | 6 | 187 | 174 | 0.25 (0.03-0.47) | 48.3% | 0.16 |  |
| HDL | 8 | 242 | 170 | 0.04 (-0.02-0.10) | 6 | 187 | 174 | -0.07 (-0.13- -0.01) | 84.1% | 0.01 |  |

**Supplementary Table 4:** Sensitivity analysis on the effect of quality of the studies.

| **Outcomes** | **High NOS studies/Total** | **PA**  **(N)** | **Control**  **(N)** | **Mean difference**  **(95% CI)** | **I_2_** |
| --- | --- | --- | --- | --- | --- |
| **Anthropometry** |  |  |  |  |  |
| Height SDS | 5/10 | 285 | 256 | 0.61 (0.38, 0.84) | 28% |
| Weight SDS | 4/7 | 154 | 117 | 0.64 (0.15, 1.13) | 60% |
| BMI SDS | 5/8 | 285 | 256 | 0.46 (0.26, 0.67) | 0% |
| BMI Z-score | 2/6 | N/A | N/A | N/A | N/A |
| **Markers of glucose metabolism** |  |  |  |  |  |
| Fasting insulin | 4/12 | 209 | 181 | 9.49 (-4.81, 23.78) | 97% |
| Fasting glucose | 5/11 | 239 | 209 | -0.03 (-0.13, 0.07) | 65% |
| HOMA-IR | 5/8 | 231 | 190 | 0.21 (-0.35, 0.77) | 97% |
| **Markers of lipid metabolism** |  |  |  |  |  |
| Cholesterol | 4/12 | 199 | 180 | -0.03 (-0.24, 0.19) | 64% |
| Triglycerides | 6/14 | 249 | 219 | 0.01 (-0.07, 0.08) | 56% |
| LDL | 4/12 | 199 | 180 | 0.02 (-0.17, 0.21) | 40% |
| HDL | 6/14 | 240 | 219 | 0.02 (-0.05, 0.10) | 36% |

**Supplementary table 5:** Sensitivity analysis on the effect of gender

| **Outcomes** | **Girls only studies/Total** | **PA**  **(N)** | **Control**  **(N)** | **Mean difference**  **(95% CI)** | **I_2_** |
| --- | --- | --- | --- | --- | --- |
| **Anthropometry** |  |  |  |  |  |
| Height SDS | 5/11 | 165 | 151 | 0.53 (0.14, 0.91) | 67% |
| Weight SDS | 4/7 | 145 | 141 | 0.86 (0.54, 1.17) | 36% |
| BMI SDS | 4/8 | 137 | 138 | 0.29 (-0.43, 1.01) | 87% |
| BMI z-score | 2/6 | N/A | N/A | N/A | N/A |
| **Markers of glucose metabolism** |  |  |  |  |  |
| Fasting insulin | 7/12 | 178 | 130 | 17.22 (-0.52, 34.96) | 80% |
| Fasting glucose | 5/11 | 142 | 106 | 0.11 (-0.19, 0.42) | 63% |
| HOMA-IR | 3/8 | 92 | 71 | 0.53 (-0.53, 1.59) | 87% |
| **Markers of lipid metabolism** |  |  |  |  |  |
| Cholesterol | 7/12 | 182 | 135 | 0.14 (-0.11, 0.39) | 69% |
| Triglycerides | 7/14 | 183 | 135 | 0.08 (-0.02, 0.18) | 44% |
| LDL | 7/12 | 182 | 135 | 0.23 (0.00, 0.46) | 61% |
| HDL | 7/14 | 182 | 135 | -0.02 (-0.09, 0.06) | 0% |
